# Appendicitis

**Published:** 1899-12-23

**Authors:** 


### APPENDICITIS.

The New York *Medical Record* for November 25th contains a very full account of the history and literature of appendicitis from the pen of Dr. George M. Edebohls, of New York. The subject has become a vast one, the bibliography which is appended to the article occupying seven and a half columns of small type, and including 376 references. The entire literature of this one subject of appendicitis, however, is said to embrace more than 2,500 journal articles, dissertations, and books! What an amount of honest labour lies buried here, drowned in a vast ocean of forgotten words!

